# Dietary magnesium and calcium intake is associated with lower risk of hearing loss in older adults: A cross-sectional study of NHANES

**DOI:** 10.3389/fnut.2023.1101764

**Published:** 2023-03-14

**Authors:** Xinmin Wei

**Affiliations:** Department of Otolaryngology, Affiliated Nanjing Jiangbei Hospital of Nantong University, Nanjing, China

**Keywords:** hearing loss, magnesium, calcium, dietary intakes, NHANES

## Abstract

**Aim:**

Dietary intake as a modifiable factor has been reported to be associated with hearing loss (HL). The relationship between magnesium (Mg) and calcium (Ca) as common dietary nutrients and HL in the elderly has rarely been reported. This study aimed to assess the association between Mg and Ca intake and HL in older adults.

**Method:**

This cross-sectional study included participants aged ≥70 years from the National Health and Nutrition Examination Survey (NHANES) 2005–2006, 2009–2010, and 2017–2018. Outcomes were low-frequency [pure-tone averages (PTAs) at 500, 1000, and 2000 Hz >25 dB] and speech-frequency (PTAs at 500, 1000, 2000, and 4,000 Hz >25 dB) HL. Multivariate logistic analysis was utilized to explore the association between dietary Mg and Ca intake and their combined intake (Ca/Mg, Ca*Mg) and HL, and was described as odds ratio (OR) and 95% confidence interval (CI).

**Results:**

A total of 1,858 participants were included, of which 1,052 (55.95%) had low-frequency HL and 1,349 (72.62%) had speech-frequency HL. Dietary Ca intakes [OR = 0.86, 95%CI: (0.74–0.99)] and Mg intakes [OR = 0.81, 95%CI: (0.68–0.95)] and Ca * Mg [OR = 0.12, 95%CI: (0.02–0.87)] were associated with lower odds of low-frequency HL after adjusting for confounders. Similar, dietary Ca intakes [OR = 0.85, 95%CI: (0.77–0.95)] and Mg intakes [OR = 0.78, 95%CI: (0.68–0.90)] and Ca * Mg [OR = 0.23, 95%CI: (0.05–0.78)] were related to lower odds of speech-frequency HL. For different levels of Mg and Ca intake, the combined intake of Ca (≥1,044 mg) and Mg (≥330 mg) was related to lower odds of low-frequency HL [OR = 0.02, 95%CI: (0.00–0.27)] and speech-frequency HL [OR = 0.44, 95%CI: (0.21–0.89)].

**Conclusion:**

Dietary intakes of Mg and Ca were associated with lower odds of HL and are a promising intervention to be further explored in older adults with HL.

## Introduction

Approximately 360 million people suffer from permanent hearing loss (HL) worldwide, accounting for 5.3% of the global population (1). The prevalence of HL is rapidly rising due to an aging population, increasingly noisy environments, and increased use of hearing devices (2). Several studies have shown that more than half of people over the age of 70 suffer from age-related HL (3, 4). HL affects elderly people’s daily life and quality of life, negatively affects their mental health (5, 6), and the health costs consumed by the prevention and treatment of HL also put pressure on socioeconomic development (7, 8). Identifying modifiable factors contributing to HL and good health management of HL are important to reduce the burden of HL in the elderly.

Age-related HL experienced by older adults usually starts with high-frequency HL and gradually affects the middle and low frequencies (9). Škerková et al. showed that hearing thresholds increase significantly with age, and people over 50 years of age can only perceive sounds at 11.2 kHz (10). Moreover, once HL has progressed into the 2–4 kHz range, speech understanding in any situation is compromised (9). It is necessary to explore the relevant factors affecting the low- and middle-frequency Hl in the elderly. Studies have shown that risk factors for HL include age, smoking, and noise exposure (11). Recently, the relationship between dietary intake and HL has received attention, and good nutritional status can help prevent or repair initial HL (12). Choi et al. found that dietary magnesium (Mg) intakes may contribute to lower hearing thresholds (13). Jung et al. reported that higher dietary Mg intake was associated with a lower incidence of HL (12). This may be due to the fact that Mg can reduce HL caused by noise-induced vasoconstriction and free radical formation (14, 15). Furthermore, calcium dysregulation is a well-recognized cause of noise HL (16), and there is an antagonistic effect between Ca and Mg (17). Ca levels affect bone density, decreased bone density is common in older adults. Curhan et al. found that osteoporosis caused by decreased bone density may increase the risk of HL (18). However, the association between dietary Mg and Ca intakes and HL at different frequency HL in the elderly remain unclear.

Therefore, the purpose of this study was to examine the association between dietary Ca and Mg intakes and the risk of HL and their interaction effects. From the perspective of improving eating habits, explore and propose health management countermeasures, to lay a theoretical foundation for hearing loss health management services.

## Methods

### Study population

Data for this cross-sectional study were extracted from the National Health and Nutrition Examination Survey (NHANES) 2005–2006, 2009–2010, and 2017–2018 (19). NHANES was conducted by the National Centers for Health Statistics (NCHS), the Centers for Disease Control and Prevention (CDC) to assess health and nutritional information for a representative sample of the U.S. civilian, noninstitutionalized U.S. population (20). The NHANES survey uses complex, multistage, probability sampling methods based on broad population distributions. Each survey cycle in the NHANES database focused on the hearing status of participants in a specific age group (21). For example, the 2007–2008 survey focused on the hearing status of adolescents aged 12–19 years, the 2009–2010 survey focused on the hearing status of adolescents aged 12–19 years and seniors aged 70 years and older, and the 2011–2012 survey focused on the hearing status of adults aged 20–69 years. After the screening, only three survey cycles of 2005–2006, 2009–2010, and 2017–2018 focused on the hearing status of participants aged 70 and above.

A total of 3,935 eligible participants aged ≥70 who received audiometric examination was in NHANES 2005–2006, 2009–2010, and 2017–2018. Participants were excluded for nonresponse or unavailable response at the audiometric examination (*n* = 7,243). Moreover, participants with missing information on dietary and zero total energy intake (*n* = 205) were excluded. Finally, 1,858 eligible participants were included in the analysis. NHANES is a publicly available dataset and was approved by the NCHS Ethics Review Board, and all participants provided written informed consent.

### Outcome variable

Audiometry examination was performed in a dedicated sound-isolating room in the Mobile Examination Center by trained examiners on participants. The outcome variables of this study were low-frequency HL and speech-frequency HL. Low-frequency HL was defined as the pure-tone averages (PTAs) at 500, 1000, and 2000 Hz >25 dB HL in either ear and speech-frequency HL was defined as PTAs at 500, 1,000, 2,000, and 4,000 Hz >25 dB HL in either ear (22). For patients with low-frequency HL, the control group was all participants except those with low-frequency HL. For patients with speech-frequency HL, the control group was all participants except those with speech-frequency HL. Among patients with both low-frequency and speech-frequency HL, the control group was participants with only low-frequency or speech-frequency HL, and participants with neither low-frequency nor speech-frequency HL.

### Intake of calcium and magnesium

Dietary Ca, Mg, vitamin C, vitamin E, and dietary supplement intakes were estimated by 24-h dietary recall interviews. Diet recall interviews require respondents to report all food and beverages (other than regular drinking water) consumed in the 24 h prior to the interview, the quantity of food reported, and a detailed description of the food (23). Food consumption data were converted to United States Department of Agriculture (USDA) standard reference codes, and food intake was linked to the USDA’s Food and Nutrient Database for Dietary Studies (FNDDS) (23, 24).

### Covariates

Demographic variables included age (70–80/ ≥80 years), sex (male/ female), race (Mexican American/ other Hispanic/ non-Hispanic White/ non-Hispanic Black/ other race), marital status (married/ widowed/ divorced or separated/never married), body mass index (BMI), education level (less than high school/high school/more than high school), physical activity, and ratio of family income to poverty (PIR). PIR was categorized as ≤130, >130 to 350%, and ≥ 350% by the federal poverty level (FPL) (25). The FPL reflects income relative to household size and was used as an indicator of socioeconomic status (26). BMI was calculated as weight (kg)/height (m)^2^. Hypertension, diabetes mellitus and cerebrovascular disease (CVD) were defined as self-reported physician diagnoses. Smoking status was defined as smoking at least 100 cigarettes in life. Loud noise exposure in past 24-h was assessed by the question of “Outside of a job, have you ever been exposed to steady loud noise or music for 5 or more hours a week? This is noise so loud that you have to raise your voice to be heard. Examples are noise from power tools, lawn mowers, farm machinery, cars, trucks, motorcycles, or loud music.” Hear protection was determined by the survey item, “How often do you wear hearing protection devices (ear plugs, ear muffs) when exposed to loud sounds or noise? (Include both job- and off-work exposures),” and it was divided into four groups: most of the time, sometimes, rarely/seldom and never. Loud noise exposure at work was participants ever had a job where exposed to loud noise for 5 or more hours a week. Physical activity was expressed as the metabolic equivalent task (MET) and calculated as follows: physical activity (met·min/week) = recommended MET × exercise time for corresponding activities (min/day) × the number of exercise days per week (day) (27). Ototoxic drug use was identified based on participants’ self-reported use of the following drugs: aminoglycosides, macrolides, antineoplastic drugs, loop diuretics, salicylates, and antimalarials (28).

### Statistical analysis

The study population was divided into two groups according to whether HL, characteristics were performed for comparison between groups. Continuous data were expressed as mean and standard error (S.E.), and the weighted t-test was used for comparison between groups. Categorical variables were described as the number and percentage [*n* (%)], and comparisons between groups used the weighted *χ*^2^ test. Missing values were filled using multiple imputations. Weighted logistic multivariate analysis was utilized to explore the association between dietary Ca intakes, dietary Mg intakes, Ca/Mg and Ca*Mg and HL. Model 1 was the crude model. Model 2 adjusted for age, sex, race/ethnicity, marital status, education level, and PIR. Model 3 adjusted for age, sex, race/ethnicity, marital status, education level, PIR, loud noise exposure in the past 24 h, loud noise exposure at work, total energy intake, vitamin C intake, vitamin E intake, physical activity, ototoxic medication use, and dietary supplement. Sensitivity analysis was performed to compare whether the results were different before and after data imputation. Moreover, Ca and Mg levels were categorized into quartiles to explore the relationship between different levels of Ca and Mg intake and HL. Odds ratio (OR) and 95% confidence interval (CI) were used to assess the association. All statistical analyzes were performed using R v. 4.20 (R Foundation for Statistical Computing, Vienna, Austria) and SAS v. 9.4 (SAS Institute, Cary, North Carolina) software. A *p*-value <0.05 (two-sided) was considered statistically significant.

## Results

### Characteristics of the study population

A total of 1,858 participants were included in the study. Characteristics of included participants were shown in Table 1. Of these participants, 1,052 (55.95%) had low-frequency HL, 1,349 (72.62%) had speech-frequency HL, and 1,046 (55.68%) had both low-and speech-frequency HL. There were 1,220 (65.32%) participants aged 70–80 years, 950 (51.37%) male participants, and 1,268 (68.10%) non-Hispanic white participants. The mean (S.E.) BMI was 28.52 (0.13) kg/m^2^. The mean (S.E.) Ca and Mg intakes were 825.84 (10.20) mg and 268.69 (3.23) mg, respectively. Dietary supplements were used by 1,503 (80.11%) participants. There were 228 (12.15%) participants who had loud noise exposure in the past 24 h and 651 (34.95%) participants who had loud noise exposure at work.

**Table 1:** Comparison of hearing loss (HL) group and non-HL group.

Variables	Total (*n* = 1858)	Low-frequency HL		*P*	Speech-frequency HL		*P*
		No (*n* = 806)	Yes (*n* = 1,052)		No (*n* = 509)	Yes (*n* = 1,349)	
Age, *n* (%)				<0.001			<0.001
70–80 years	1,220 (65.32)	650 (80.67)	570 (54.46)		436 (86.30)	784 (58.36)	
≥80 years	638 (34.68)	156 (19.33)	482 (45.54)		73 (13.70)	565 (41.64)	
Race/ethnicity, *n* (%)				<0.001			<0.001
Mexican American	148 (7.78)	63 (7.45)	85 (7.69)		45 (8.65)	103 (7.18)	
Other Hispanic	72 (4.04)	41 (5.01)	31 (2.95)		25 (4.67)	47 (3.55)	
Non-Hispanic White	1,268 (68.10)	506 (63.67)	762 (73.80)		288 (57.62)	980 (73.76)	
Non-Hispanic Black	278 (14.92)	155 (18.82)	123 (11.30)		118 (22.94)	160 (11.47)	
Other race	92 (5.15)	41 (5.06)	51 (4.27)		33 (6.13)	59 (4.04)	
Sex, *n* (%)				0.059			<0.001
Male	908 (48.63)	378 (46.08)	530 (50.18)		195 (37.88)	713 (52.33)	
Female	950 (51.37)	428 (53.92)	522 (49.82)		314 (62.12)	636 (47.67)	
Marital status, *n* (%)				<0.001			0.022
Married	1,001 (53.65)	452 (56.01)	549 (53.51)		270 (53.15)	731 (55.16)	
Widowed	572 (30.67)	205 (25.42)	367 (33.34)		139 (27.24)	433 (30.84)	
Divorced/separated	220 (12.06)	118 (14.67)	102 (9.81)		78 (15.39)	142 (10.65)	
Never married	65 (3.62)	31 (3.90)	34 (3.33)		22 (4.22)	43 (3.34)	
BMI, kg/m^2^, Mean (S.E)	28.52 (0.13)	28.80 (0.22)	28.37 (0.15)	0.090	28.63 (0.32)	28.54 (0.14)	0.809
Education level, *n* (%)				<0.001			<0.001
Less than high school	540 (29.13)	201 (24.07)	339 (31.65)		113 (21.57)	427 (30.86)	
High school	491 (26.49)	208 (25.71)	281 (26.63)		125 (24.53)	364 (26.87)	
More than high school	827 (44.38)	397 (50.21)	432 (41.72)		271 (53.89)	558 (42.28)	
PIR, *n* (%)				0.047			0.229
<130	432 (23.05)	204 (24.23)	312 (28.33)		129 (23.88)	387 (27.52)	
130–350%	906 (49.08)	373 (47.49)	499 (48.27)		237 (47.92)	635 (47.93)	
≥350%	520 (27.87)	229 (28.28)	241 (23.39)		143 (28.20)	327 (24.55)	
Smoking, *n* (%)				0.727			0.533
Yes	968 (51.89)	428 (52.53)	540 (51.61)		260 (50.83)	708 (52.46)	
No	890 (48.11)	378 (47.47)	512 (48.39)		249 (49.17)	641 (47.54)	
Physical activity, MET·min, Mean (S.E)	1486.64 (111.58)	1701.22 (209.16)	1328.44 (139.84)	0.159	1542.94 (166.27)	1473.71 (139.16)	0.734
Diabetes mellitus, *n* (%)				0.832			0.981
No	1,414 (75.74)	609 (75.75)	805 (76.18)		387 (75.95)	1,027 (76.00)	
Yes	444 (24.26)	197 (24.25)	247 (23.82)		122 (24.05)	322 (24.00)	
Hypertension, *n* (%)				0.432			0.625
No	452 (24.26)	199 (25.14)	254 (23.60)		126 (25.04)	327 (23.99)	
Yes	1,406 (75.74)	607 (74.86)	798 (76.40)		383 (74.96)	1,022 (76.01)	
CVD, *n* (%)				0.146			0.444
No	442 (23.72)	201 (25.27)	242 (22.19)		126 (24.87)	317 (23.05)	
Yes	1,416 (76.28)	605 (74.73)	810 (77.81)		383 (75.13)	1,032 (76.95)	
Loud noise exposure in past 24-h, *n* (%)				<0.001			<0.001
Yes	228 (12.15)	81 (10.31)	147 (13.97)		40 (7.94)	188 (14.02)	
No	1,630 (87.85)	725 (89.69)	905 (86.03)		469 (92.06)	1,161 (85.98)	
Loud noise exposure at work, *n* (%)				0.052			0.004
Yes	651 (34.95)	254 (31.74)	400 (37.67)		139 (27.75)	515 (37.82)	
No	1,207 (65.05)	552 (68.26)	652 (62.33)		370 (72.25)	834 (62.18)	
Hear protection, *n* (%)				<0.001			<0.001
Most of the time	344 (18.58)	124 (15.42)	184 (17.55)		75 (14.39)	233 (17.45)	
Sometimes	141 (7.55)	58 (7.31)	69 (6.30)		33 (6.58)	94 (6.81)	
Rarely/seldom	1,130 (60.44)	400 (48.75)	603 (57.48)		249 (48.49)	754 (55.58)	
Never	243 (13.42)	224 (28.51)	196 (18.67)		152 (30.55)	268 (20.16)	
Calcium intake, mg, Mean (S.E)	825.84 (10.20)	846.84 (17.04)	817.59 (11.87)	0.152	840.98 (20.15)	826.52 (10.38)	0.477
Magnesium intake, mg, Mean (S.E)	268.69 (3.23)	279.39 (4.77)	261.62 (4.01)	0.004	280.11 (6.45)	265.43 (3.29)	0.029
Vitamin C intake, mg, Mean (S.E)	85.46 (2.21)	86.03 (2.98)	86.35 (2.38)	0.907	86.03 (3.84)	86.28 (2.20)	0.941
Vitamin E intake, mg, Mean (S.E)	7.88 (0.16)	8.16 (0.26)	7.63 (0.23)	0.159	7.92 (0.29)	7.84 (0.19)	0.812
Total energy, kcal, Mean (S.E)	1742.34 (18.22)	1789.74 (26.35)	1712.71 (23.11)	0.014	1761.09 (27.05)	1741.20 (20.71)	0.456
Dietary supplements, *n* (%)				0.088			0.034
Yes	1,503 (80.11)	463 (59.75)	557 (54.87)		300 (61.46)	720 (55.34)	
No	355 (19.89)	343 (40.25)	495 (45.13)		209 (38.54)	629 (44.66)	
Ototoxic medication use, *n* (%)				0.043			0.018
No	1,573 (84.35)	696 (86.19)	877 (82.91)		450 (88.28)	1,123 (82.87)	
Yes	285 (15.65)	110 (13.81)	175 (17.09)		59 (11.72)	226 (17.13)	

PIR: ratio of family income to poverty; BMI: body mass index; CVD: cerebrovascular disease.

### Comparison between the HL group and the non-HL group

Comparing the differences between low-frequency HL and non-low-frequency HL groups, the results showed that the two groups were significant in age (*p* < 0.001), race/ethnicity (*p* < 0.001), marital status (*p* < 0.001), education level (*p* < 0.001), PIR (*p* = 0.047), loud noise exposure in the past 24 h (*p* < 0.001), hear protection when noise exposure (*p* < 0.001), total energy intake (*p* = 0.014), and ototoxic medication use (*p* = 0.043; Table 1). In the speech-frequency HL and non-speech-frequency HL groups, there were significant differences in age (*p* < 0.001), race/ethnicity (*p* < 0.001), sex (*p* < 0.001), marital status (*p* = 0.022), educational level (*p* < 0.001), loud noise exposure in the past 24 h (*p* < 0.001), loud noise exposure at work (*p* = 0.004), hear protection when noise exposure (*p* < 0.001), dietary supplements (*p* = 0.034), and ototoxic medication use (*p* = 0.018).

### Association between dietary ca and mg intake and HL

Table 2 reports the effects of dietary Ca and Mg intake and their combination (Ca/Mg and Ca * Mg) on HL. Dietary Ca intakes [OR = 0.86, 95%CI: (0.75–0.99)] and Mg intakes [OR = 0.77, 95%CI: (0.67–0.90)] and Ca * Mg [OR = 0.22, 95%CI: (0.05–0.86)] were associated with lower odds of low-frequency HL. After adjusting for age, sex, race/ethnicity, marital status, education level, PIR, loud noise exposure in the past 24 h, loud noise exposure at work, total energy intake, vitamin C intake, vitamin E intake, physical activity, ototoxic medication, and dietary supplements (model 3), dietary Ca intakes [OR = 0.86, 95%CI: (0.74–0.99)] and Mg intakes [OR = 0.81, 95%CI: (0.68–0.95)] and Ca * Mg [OR = 0.12, 95%CI: (0.02–0.87)] were still associated with lower odds of low-frequency HL. In the analysis of speech-frequency HL, dietary Ca intakes [OR = 0.85, 95%CI: (0.77–0.95)] and Mg intakes [OR = 0.78, 95%CI: (0.68–0.90)] and Ca * Mg [OR = 0.23, 95%CI: (0.05–0.78)] were related to lower odds of speech-frequency HL after adjusting for all confounders. Among participants with both low- and speech-frequency HL, dietary Ca intakes [OR = 0.85, 95%CI: (0.77–0.95)] and Mg intakes [OR = 0.78, 95%CI: (0.68–0.90)] and Ca * Mg [OR = 0.23, 95%CI: (0.05–0.78)] were also related to lower odds of low- and speech-frequency HL after adjusting for all confounders. In the sensitivity analysis (Supplementary Table S1), there was no significant difference in the results before and after data imputation.

**Table 2:** Association of dietary calcium (Ca) and magnesium (Mg) intakes with hearing loss (HL).

Outcomes	Model 1		Model 2		Model 3	
	OR (95%CI)	*P*	OR (95%CI)	*P*	OR (95%CI)	*P*
Low-frequency HL						
Ca	0.86 (0.75–0.99)	0.032	0.85 (0.74–0.97)	0.020	0.86 (0.74–0.99)	0.035
Mg	0.77 (0.67–0.90)	<0.001	0.80 (0.69–0.94)	0.008	0.81 (0.68–0.95)	0.011
Ca/Mg	1.09 (0.93–1.28)	0.258	1.03 (0.89–1.19)	0.710	1.02 (0.88–1.18)	0.809
Ca * Mg	0.22 (0.05–0.86)	0.035	0.25 (0.04–0.93)	0.044	0.12 (0.02–0.87)	0.041
Speech-frequency HL						
Ca	0.89 (0.77–1.03)	0.120	0.91 (0.84–0.99)	0.032	0.85 (0.77–0.95)	0.004
Mg	0.81 (0.69–0.94)	0.007	0.85 (0.77–0.95)	0.003	0.78 (0.68–0.90)	0.001
Ca/Mg	1.14 (0.99–1.32)	0.073	1.06 (0.95–1.19)	0.265	1.04 (0.93–1.16)	0.470
Ca * Mg	0.11 (0.02–0.76)	0.030	0.11 (0.03–0.75)	0.036	0.23 (0.05–0.78)	0.041
Low- and speech-frequency HL						
Ca	0.89 (0.81–0.97)	0.010	0.83 (0.73–0.95)	0.006	0.83 (0.72–0.95)	0.010
Mg	0.82 (0.75–0.91)	<0.001	0.82 (0.72–0.95)	0.008	0.83 (0.76–0.91)	<0.001
Ca/Mg	1.06 (0.97–1.17)	0.206	1.00 (0.85–1.19)	0.973	0.96 (0.88–1.05)	0.358
Ca * Mg	0.20 (0.05–0.89)	0.040	0.26 (0.06–0.95)	0.048	0.19 (0.04–0.79)	0.027

OR: odds ratio; CI: confidence interval. Model 1: the crude model. Model 2: adjusted for age, sex, race/ethnicity, marital status, education level, and PIR. Model 3: adjusted for age, sex, race/ethnicity, marital status, education level, PIR, loud noise exposure in the past 24 h, loud noise exposure at work, total energy intake, vitamin C intake, vitamin E intake, physical activity, ototoxic medication, and dietary supplement.

### Association between different levels of ca and mg intake and HL

Compared with Ca intake <545 mg, only participants with Ca intake ≥1,044 mg [OR = 0.61, 95%CI: (0.46–0.81)] were related to lower odds of low-frequency HL after adjusting for all confounders. Compared with Mg intake <190 mg, only participants with Mg intake ≥330 mg [OR = 0.67, 95%CI: (0.46–0.99)] were related to lower odds of low-frequency HL after adjusting for all confounders (Supplement Table S2). Similar, only participants with Ca intake ≥1,044 mg [OR = 0.59, 95%CI: (0.38–0.90)] or Mg intake ≥330 mg [OR = 0.64, 95%CI: (0.41–0.99)] were associated with lower odds of speech-frequency HL after adjusting for all confounders. Table 3 shows the effect of combined intake of different levels of Ca and Mg on HL. Combined intake of Ca (545-756 mg) and Mg (≥330 mg) [OR = 0.14, 95%CI: (0.02–0.74)] or Ca (≥1,044 mg) and Mg (≥330 mg) [OR = 0.02, 95%CI: (0.00–0.27)] was related to lower odds of low-frequency HL. Combined intake of Ca (545-756 mg) and Mg (≥330 mg) [OR = 0.34, 95%CI: (0.12–1.00)] or Ca (≥1,044 mg) and Mg (252-330 mg) [OR = 0.37, 95%CI: (0.17–0.84)] or Ca (≥1,044 mg) and Mg (≥330 mg) [OR = 0.44, 95%CI: (0.21–0.89)] was associated with lower odds of speech-frequency HL. Similar results were observed in participants with both low- and speech-frequency HL.

**Table 3:** Association between combined intake of different levels of calcium (Ca) and magnesium (Mg) and hearing loss (HL).

Outcomes	Levels	Model 2		Model 3	
		OR (95%CI)	*P*	OR (95%CI)	*P*
Low-frequency HL	Ca (<545 mg) and Mg (<190 mg)	Ref		Ref	
	Ca (545-756 mg) and Mg (190-252 mg)	1.37 (0.89–2.10)	0.149	0.66 (0.24–1.77)	0.405
	Ca (545-756 mg) and Mg (252-330 mg)	0.92 (0.47–1.81)	0.815	1.23 (0.37–4.13)	0.734
	Ca (545-756 mg) and Mg (≥330 mg)	0.47 (0.20–1.10)	0.082	0.14 (0.02–0.74)	0.021
	Ca (756-1,044 mg) and Mg (190-252 mg)	0.92 (0.54–1.58)	0.772	1.44 (0.48–4.30)	0.510
	Ca (756-1,044 mg) and Mg (252-330 mg)	0.76 (0.48–1.21)	0.245	3.37 (0.99–11.47)	0.052
	Ca (756-1,044 mg) and Mg (≥330 mg)	0.69 (0.41–1.15)	0.157	0.61 (0.11–3.33)	0.571
	Ca (≥1,044 mg) and Mg (190-252 mg)	1.51 (0.54–4.17)	0.432	0.07 (0.00–1.36)	0.080
	Ca (≥1,044 mg) and Mg (252-330 mg)	0.59 (0.35–0.99)	0.049	0.08 (0.00–1.44)	0.087
	Ca (≥1,044 mg) and Mg (≥330 mg)	0.61 (0.40–0.93)	0.020	0.02 (0.00–0.27)	0.004
Speech-frequency HL	Ca (<545 mg) and Mg (<190 mg)	Ref		Ref	
	Ca (545-756 mg) and Mg (190-252 mg)	1.52 (0.68–3.40)	0.303	1.31 (0.58–2.94)	0.515
	Ca (545-756 mg) and Mg (252-330 mg)	0.71 (0.28–1.79)	0.466	0.67 (0.25–1.81)	0.429
	Ca (545-756 mg) and Mg (≥330 mg)	0.36 (0.13–0.98)	0.045	0.34 (0.12–1.00)	0.050
	Ca (756-1,044 mg) and Mg (190-252 mg)	0.58 (0.27–1.23)	0.156	0.62 (0.27–1.43)	0.262
	Ca (756-1,044 mg) and Mg (252-330 mg)	1.15 (0.52–2.55)	0.739	1.10 (0.50–2.43)	0.809
	Ca (756-1,044 mg) and Mg (≥330 mg)	0.58 (0.22–1.54)	0.285	0.52 (0.16–1.66)	0.270
	Ca (≥1,044 mg) and Mg (190-252 mg)	1.47 (0.40–5.34)	0.561	1.37 (0.38–4.90)	0.601
	Ca (≥1,044 mg) and Mg (252-330 mg)	0.41 (0.19–0.88)	0.023	0.37 (0.17–0.84)	0.018
	Ca (≥1,044 mg) and Mg (≥330 mg)	0.47 (0.28–0.80)	0.005	0.44 (0.21–0.89)	0.023
Low- and speech-frequency HL	Ca (<545 mg) and Mg (<190 mg)	Ref		Ref	
	Ca (545-756 mg) and Mg (190-252 mg)	0.68 (0.27–1.74)	0.425	0.59 (0.23–1.51)	0.272
	Ca (545-756 mg) and Mg (252-330 mg)	1.33 (0.42–4.19)	0.623	1.21 (0.37–3.95)	0.753
	Ca (545-756 mg) and Mg (≥330 mg)	0.18 (0.03–1.02)	0.053	0.16 (0.03–0.87)	0.034
	Ca (756-1,044 mg) and Mg (190-252 mg)	1.64 (0.55–4.85)	0.374	1.57 (0.53–4.69)	0.418
	Ca (756-1,044 mg) and Mg (252-330 mg)	3.88 (1.25–12.11)	0.019	3.63 (1.09–12.05)	0.035
	Ca (756-1,044 mg) and Mg (≥330 mg)	0.92 (0.17–4.92)	0.926	0.72 (0.13–3.93)	0.707
	Ca (≥1,044 mg) and Mg (190-252 mg)	0.05 (0.00–0.92)	0.438	0.06 (0.00–0.92)	0.044
	Ca (≥1,044 mg) and Mg (252-330 mg)	0.08 (0.01–1.24)	0.070	0.08 (0.01–1.22)	0.069
	Ca (≥1,044 mg) and Mg (≥330 mg)	0.02 (0.00–0.31)	0.005	0.02 (0.00–0.26)	0.003

OR: odds ratio; CI: confidence interval; Ref, reference. Model 2: adjusted for age, sex, race/ethnicity, marital status, educational level, and PIR. Model 3: adjusted for age, sex, race/ethnicity, marital status, educational level, PIR, loud noise exposure in the past 24 h, loud noise exposure at work, total energy intake, vitamin C intake, vitamin E intake, physical activity, ototoxic medication, and dietary supplements.

## Discussion

HL is one of the chronic non-communicable diseases that can affect people’s quality of life. HL not only has a negative impact on daily life and mental health, but its prevention and treatment costs also bring pressure to social and economic development. The present study found that dietary Mg and Ca intake and their combined intake were inversely associated with the odds of low- and speech-frequency HL. Among different levels of Mg and Ca intake, Mg levels ≥330 mg combined with Ca levels ≥1,044 mg were related to lower odds of low- and speech-frequency HL.

HL at different frequencies can be divided into low-frequency, speech-frequency, and high-frequency HL (29). High-frequency audiometry is mainly used for early detection of hearing loss. Age-related HL experienced by older adults usually starts with high-frequency HL and gradually affects the middle and low frequencies and audiometry in the 2–4 kHz range is important for the assessment of speech comprehension (9). Therefore, the current study focused on low- and speech-frequency HL in older adults aged ≥70 years. Our results showed that the prevalence of high-frequency hearing loss in participants aged ≥70 years was 95.07%, which was why we chose low- and speech-frequency HL as the outcome of our study. Many studies have reported the association between dietary Mg intake and HL. The cross-sectional study of Choi et al. showed that dietary Mg intake was associated with lower risks of HL (13), which was consistent with our results. Spankovich et al. suggested that higher Mg intake was significantly associated with better pure tone thresholds in the populations of Sydney Australia (30). Several studies have pointed out that oxidative stress was related to HL (14). The relationship between antioxidants and HL has been confirmed in many animal studies, the formation of free radicals in the inner ear is a key factor in HL, and Mg can reduce the vasoconstriction caused by the formation of free radicals, thereby protecting hearing (14, 15). These studies provide a scientific basis to support our epidemiological findings. Weyh et al. reported the role of minerals such as Mg in immune system function and regulation of inflammation (31). Therefore, dietary Mg intake is a promising intervention for elderly patients with HL and further studies are needed.

Currently, few studies were studied on the association between dietary Ca intakes and HL. The present study found an inversely associations between dietary Ca intake and low- and speech-frequency HL. Moreover, the product of dietary Ca and dietary Mg was found to be associated with lower odds of low-frequency and speech-frequency HL after adjusting for all confounders (including vitamin C, vitamin E, physical activity). To the best of our knowledge, this study was the first to analyze the association between combined intake of Ca and Mg and HL. The present study may provide epidemiological evidence for the relationship between Ca and Mg intake and HL in older adults. Dietary Ca and Mg intake is a promising intervention to be further explored in older adults with HL. However, the specific mechanism by which Mg and Ca affect HL is still unknown, and the mechanisms that can be speculated are as follows. Mg is a Ca antagonist, and Mg antagonizes Ca when absorbed in the small intestine, and chronic low levels of Ca may be related to underlying Mg deficiency (32). Mg could block the excessive release of Ca in hair cells and the cochlear vasculature, limits cellular energy consumption and induces arteriole vasodilation (33). Through this mechanism, Mg could inhibit ischemia caused by hearing damage (33). Hypomagnesemia disrupted Ca homeostasis, thereby enhancing the pro-inflammatory effects of Mg deficiency (34). Inflammatory effects are generally associated with free radicals and thus have an effect on HL (14, 35).

Several limitations of this study should be considered. First, we only used three surveys of 2005–2006, 2009–2010, and 2017–2018, which may lead to small sample size and reduced statistical power due to the different ages of participants who participated in the audiometry examination in different years in the NHANES database. Second, the cross-sectional study design of this study could not establish a causal relationship between dietary Ca and Mg intake and HL. Third, the dietary data was from a dietary recall survey, and there may be participants with cognitive impairments among the surveyed elderly, which may cause a certain recall bias. Prospective large-scale studies are needed to further explore the relationship between dietary Ca and Ma and HL.

## Conclusion

This study found that dietary Mg and Ca intake and their combination were inversely associated with low- and speech-frequency HL. Identification of modifiable factors affecting elderly patients with HL plays an important role in patient management and prevention. Dietary Mg and Ca intake is a promising intervention to be further explored in older adults with HL.

## Data availability statement

Publicly available datasets were analyzed in this study. This data can be found here NHANES, https://wwwn.cdc.gov/nchs/nhanes/.

## Ethics statement

Ethical approval was not provided for this study on human participants because NHANES is a publicly available data set. The patients/participants provided their written informed consent to participate in this study.

## Author contributions

XW designed the study, wrote the manuscript, collected, analyzed and interpreted the data, and critically reviewed, edited and approved the manuscript.

## Conflict of interest

The author declares that the research was conducted in the absence of any commercial or financial relationships that could be construed as a potential conflict of interest.

## Publisher’s note

All claims expressed in this article are solely those of the authors and do not necessarily represent those of their affiliated organizations, or those of the publisher, the editors and the reviewers. Any product that may be evaluated in this article, or claim that may be made by its manufacturer, is not guaranteed or endorsed by the publisher.
