# Supplementary material for: Dietary magnesium and calcium intake is associated with lower risk of hearing loss in older adults: A cross-sectional study of NHANES
Source: Front Nutr. 2023 Mar 14;10:1101764. doi: 10.3389/fnut.2023.1101764 (PMC10043168; doi:10.3389/fnut.2023.1101764)
Supplement: Supplementary file 1 [file Table_1.DOCX]

**Supplementary Table 1** Sensitivity analysis before data imputation

|  | Model 1 | | Model 2 | | Model 3 | |
| --- | --- | --- | --- | --- | --- | --- |
|  | OR (95%CI) | *P* | OR (95%CI) | *P* | OR (95%CI) | *P* |
| Low-frequency HL |  |  |  |  |  |  |
| Ca | 0.86 (0.75-0.99) | 0.032 | 0.83 (0.72-0.96) | 0.011 | 0.81 (0.69-0.96) | 0.016 |
| Mg | 0.77 (0.67-0.90) | <0.001 | 0.81 (0.68-0.96) | 0.016 | 0.80 (0.68-0.95) | 0.013 |
| Ca/Mg | 1.09 (0.93-1.28) | 0.258 | 1.00 (0.86-1.15) | 0.966 | 1.04 (0.86-1.26) | 0.693 |
| Ca * Mg | 0.22 (0.05-0.86) | 0.035 | 0.10 (0.02-0.51) | 0.006 | 0.09 (0.02-0.46) | 0.004 |
| Speech-frequency HL |  |  |  |  |  |  |
| Ca | 0.89 (0.77-1.03) | 0.120 | 0.87 (0.80-0.94) | 0.002 | 0.82 (0.72-0.93) | 0.003 |
| Mg | 0.81 (0.69-0.94) | 0.007 | 0.83 (0.75-0.92) | 0.001 | 0.74 (0.63-0.88) | <0.001 |
| Ca/Mg | 1.14 (0.99-1.32) | 0.073 | 1.04 (0.92-1.17) | 0.573 | 1.04 (0.91-1.19) | 0.575 |
| Ca * Mg | 0.11 (0.02-0.76) | 0.030 | 0.11 (0.01-0.82) | 0.032 | 0.08 (0.01 - 0.99) | 0.049 |
| Low- and speech-frequency HL |  |  |  |  |  |  |
| Ca | 0.89 (0.81-0.97) | 0.010 | 0.83 (0.72-0.95) | 0.010 | 0.82 (0.70-0.96) | 0.015 |
| Mg | 0.82 (0.75-0.91) | <0.001 | 0.81 (0.69-0.96) | 0.013 | 0.82 (0.69-0.96) | 0.017 |
| Ca/Mg | 1.06 (0.97-1.17) | 0.206 | 1.01 (0.85-1.20) | 0.886 | 1.02 (0.85-1.23) | 0.821 |
| Ca * Mg | 0.20 (0.05-0.89) | 0.040 | 0.09 (0.01-0.51) | 0.007 | 0.07 (0.01-0.49) | 0.007 |

OR: odds ratio; CI: confidence interval;

Model 1: the crude model.

Model 2: adjusted for age, sex, race/ethnicity, marital status, education level, and PIR.

Model 3: adjusted for age, sex, race/ethnicity, marital status, education level, PIR, loud noise exposure in the past 24 hours, loud noise exposure at work, total energy intake, vitamin C intake, vitamin E intake, physical activity, ototoxic medication, and dietary supplement.

**Supplementary Table 2** Association between different levels of calcium (Ca) and magnesium (Mg) intake and hearing loss (HL).

| Outcomes | Levels | Model 1 | | Model 2 | | Model 3 | |
| --- | --- | --- | --- | --- | --- | --- | --- |
|  |  | OR (95%CI) | *P* | OR (95%CI) | *P* | OR (95%CI) | *P* |
| Low-frequency HL | **Ca** |  |  |  |  |  |  |
|  | <545 mg | Ref |  | Ref |  | Ref |  |
|  | 545~756 mg | 0.91 (0.68-1.22) | 0.524 | 0.95 (0.70-1.29) | 0.755 | 0.96 (0.71-1.30) | 0.781 |
|  | 756~1044 mg | 0.86 (0.66-1.11) | 0.239 | 0.83 (0.64-1.08) | 0.157 | 0.80 (0.62-1.04) | 0.100 |
|  | ≥1044 mg | 0.69 (0.52-0.91) | 0.011 | 0.67 (0.51-0.87) | 0.004 | 0.61 (0.46-0.81) | <0.001 |
|  | **Mg** |  |  |  |  |  |  |
|  | <190 mg | Ref |  | Ref |  | Ref |  |
|  | 190~252 mg | 1.22 (0.92-1.64) | 0.168 | 1.29 (0.94-1.77) | 0.110 | 1.27 (0.91-1.79) | 0.158 |
|  | 252~330 mg | 0.74 (0.59-0.94) | 0.015 | 0.78 (0.58-1.05) | 0.102 | 0.74 (0.53-1.03) | 0.078 |
|  | ≥330 mg | 0.64 (0.48-0.85) | 0.003 | 0.71 (0.52-0.99) | 0.041 | 0.67 (0.46-0.99) | 0.046 |
|  | **Ca/Mg** |  |  |  |  |  |  |
|  | <2.31 | Ref |  | Ref |  | Ref |  |
|  | 2.31~3.02 | 1.08 (0.75-1.54) | 0.688 | 1.08 (0.75-1.55) | 0.687 | 1.10 (0.77-1.58) | 0.583 |
|  | 3.03~3.86 | 1.10 (0.84-1.45) | 0.478 | 0.98 (0.75-1.27) | 0.879 | 0.94 (0.71-1.23) | 0.629 |
|  | ≥3.86 | 1.20 (0.89-1.62) | 0.220 | 1.00 (0.73-1.34) | 0.963 | 0.96 (0.72-1.29) | 0.796 |
| Speech-frequency HL | **Ca** |  |  |  |  |  |  |
|  | <545 mg | Ref |  | Ref |  | Ref |  |
|  | 545~756 mg | 0.75 (0.44-1.26) | 0.270 | 0.67 (0.38-1.18) | 0.164 | 0.87 (0.54-1.38) | 0.535 |
|  | 756~1044 mg | 0.58 (0.30-1.11) | 0.097 | 0.53 (0.27-1.04) | 0.065 | 0.68 (0.44-1.04) | 0.077 |
|  | ≥1044 mg | 0.49 (0.27-0.86) | 0.015 | 0.40 (0.22-0.73) | 0.003 | 0.59 (0.38-0.90) | 0.016 |
|  | **Mg** |  |  |  |  |  |  |
|  | <190 mg | Ref |  | Ref |  | Ref |  |
|  | 190~252 mg | 1.19 (0.66-2.12) | 0.559 | 1.26 (0.67-2.38) | 0.467 | 1.23 (0.82-1.86) | 0.306 |
|  | 252~330 mg | 0.76 (0.40-1.42) | 0.379 | 0.83 (0.43-1.59) | 0.560 | 0.72 (0.49-1.05) | 0.088 |
|  | ≥330 mg | 0.50 (0.27-0.93) | 0.030 | 0.56 (0.32-0.96) | 0.036 | 0.64 (0.41-0.99) | 0.047 |
|  | **Ca/Mg** |  |  |  |  |  |  |
|  | <2.31 | Ref |  | Ref |  | Ref |  |
|  | 2.31~3.02 | 1.27 (0.73-2.22) | 0.383 | 1.24 (0.70-2.22) | 0.454 | 1.24 (0.80-1.93) | 0.325 |
|  | 3.03~3.86 | 1.00 (0.57-1.75) | 0.986 | 0.83 (0.45-1.51) | 0.531 | 1.02 (0.66-1.57) | 0.943 |
|  | ≥3.86 | 0.97 (0.48-1.95) | 0.930 | 0.73 (0.35-1.50) | 0.379 | 0.98 (0.61-1.56) | 0.920 |
| Low- and speech-frequency HL | **Ca** |  |  |  |  |  |  |
|  | <545 mg | Ref |  | Ref |  | Ref |  |
|  | 545~756 mg | 0.94 (0.70-1.28) | 0.710 | 0.88 (0.56-1.39) | 0.578 | 0.88 (0.55-1.42) | 0.597 |
|  | 756~1044 mg | 0.88 (0.67-1.17) | 0.378 | 0.69 (0.45-1.05) | 0.081 | 0.72 (0.47-1.11) | 0.133 |
|  | ≥1044 mg | 0.70 (0.53-0.92) | 0.011 | 0.59 (0.39-0.90) | 0.016 | 0.60 (0.39-0.93) | 0.023 |
|  | **Mg** |  |  |  |  |  |  |
|  | <190 mg | Ref |  | Ref |  | Ref |  |
|  | 190~252 mg | 1.25 (0.94-1.67) | 0.123 | 1.23 (0.82-1.85) | 0.313 | 1.19 (0.79-1.79) | 0.403 |
|  | 252~330 mg | 0.80 (0.62-1.03) | 0.081 | 0.72 (0.49-1.06) | 0.097 | 0.70 (0.47-1.04) | 0.073 |
|  | ≥330 mg | 0.68 (0.51-0.92) | 0.014 | 0.64 (0.41-0.99) | 0.049 | 0.63 (0.41-0.98) | 0.043 |
|  | **Ca/Mg** |  |  |  |  |  |  |
|  | <2.31 | Ref |  | Ref |  | Ref |  |
|  | 2.31~3.02 | 1.07 (0.75-1.53) | 0.703 | 1.27 (0.83-1.96) | 0.268 | 1.25 (0.81-1.93) | 0.307 |
|  | 3.03~3.86 | 1.09 (0.82-1.45) | 0.537 | 1.01 (0.66-1.55) | 0.945 | 0.98 (0.62-1.52) | 0.912 |
|  | ≥3.86 | 1.14 (0.84-1.54) | 0.393 | 1.00 (0.62-1.58) | 0.955 | 1.01 (0.64-1.60) | 0.956 |

OR: odds ratio; CI: confidence interval;

Model 1: the crude model.

Model 2: adjusted for age, sex, race/ethnicity, marital status, education level, and PIR.

Model 3: adjusted for age, sex, race/ethnicity, marital status, education level, PIR, loud noise exposure in the past 24 hours, loud noise exposure at work, total energy intake, vitamin C intake, vitamin E intake, physical activity, ototoxic medication, and dietary supplement.
